# Supplementary material for: Comparative multiomics analysis of cell physiological state after culture in a basket bioreactor
Source: Sci Rep. 2022 Nov 23;12:20161. doi: 10.1038/s41598-022-24687-4 (PMC9686226; doi:10.1038/s41598-022-24687-4)
Supplement: Supplementary file 1 — Supplementary Information 1. [file 41598_2022_24687_MOESM1_ESM.zip › raw data/Transcriptomic raw data/6.Enrichment/2.KEGG/TE1vsTC1/ALL/src/csab04010.html]

csab04010


- csab:103216504

- Up regulated genes

103216504(2.06416519193894)

- csab:103235528

- Down regulated genes

103235528(-1.85219472562622)

- csab:103225530

- Up regulated genes

103225530(1.52855282236579)
- csab:103246479

- Down regulated genes

103246479(-1.5490263767958)

- csab:103217090

- Up regulated genes

103217090(1.81328286766627)

- csab:103238335

- Up regulated genes

103238335(2.31055276582108)

- csab:103216504

- Up regulated genes

103216504(2.06416519193894)

- csab:103225290

- Down regulated genes

103225290(-1.66879889448618)

- csab:103224652

- Up regulated genes

103224652(1.58918989505374)

- csab:103241278

- Down regulated genes

103241278(-4.29901042433954)

- csab:103223677

- Down regulated genes

103223677(-2.64469587337696)

- csab:103237430

- Up regulated genes

103237430(1.35562492669487)

- csab:103235805

- Up regulated genes

103235805(2.54406016949455)
- csab:103235804

- Up regulated genes

103235804(2.53749812915487)

Close
